# Beyond bar charts

**DOI:** 10.1186/s12915-015-0169-6

**Published:** 2015-08-06

**Authors:** Emma Saxon

**Affiliations:** BMC Biology, BioMed Central, 236 Gray’s Inn Road, London, WC1X 8HB UK

## Abstract

Probiotic treatments are thought to increase the levels of commensal bacterial species that populate the human gut, causing no harm to their host and playing an important role in maintaining gut health. This study is an investigation of the effect of a probiotic treatment on the level of a known commensal bacterium in the guts of healthy human subjects, which was significantly increased with probiotic treatment compared with a control. The authors concluded that the probiotic may thus help to promote gut health.

## Commentary

**Fig. 1.**
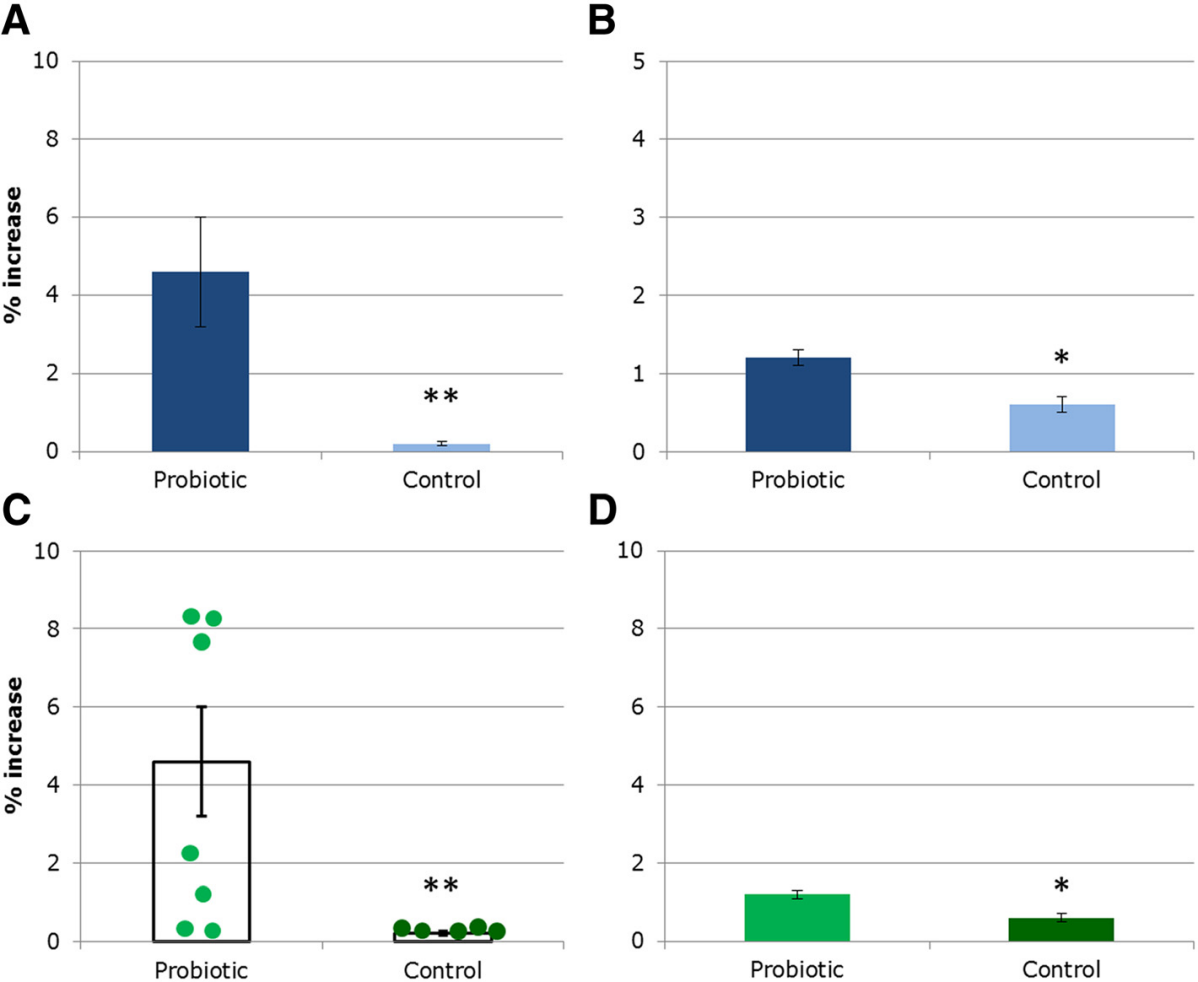
Percentage increase of a commensal species in the human gut bacterial population in response to probiotic treatment. **a**, **c** Summaries of data from the initial published experiment (n = 7/5 in treatment/control groups); **b**, **d** Summaries of data from a repeat of this experiment (n = 25 in each group). Student’s *t*-test; *p < 0.05, **p < 0.01. Error bars represent ± 1 standard deviation

Bar charts are often used to display results, but can misrepresent or obscure patterns in the data. The original data from this study are summarized in Fig. 1a, which appears to support the authors’ conclusion that the probiotic significantly increased the proportion of the commensal species in the gut bacterial community (Student’s *t*-test *p* < 0.01). These results were subsequently disputed on publication of conflicting evidence from another research group. In response, the authors of this study conducted their assay with a larger sample size, shown in Fig. 1b, to demonstrate that their original results were replicable. Indeed, on first glance the data also show a significant trend in the second assay (Student’s *t*-test *p* < 0.05), although the difference between probiotic and control treatments appears slightly smaller.

However, when the data are displayed in a scatter plot, showing the response of each individual to the probiotic treatment in the original study (Fig. 1c), a more complex picture emerges. It turns out that the high mean increase in gut commensal bacteria is due to the three individuals that make up almost half of the sample, for which this value was particularly large, and much smaller increases occurred in the other probiotic-treated mice. The smaller increase seen in the second assay may, therefore, be a more accurate reflection of the response of the majority; but without seeing the individual points it is impossible to tell. A Shapiro-Wilk test demonstrates that the data points in Fig. 1c are not normally distributed, but a normal distribution is assumed by the *t*-test used here; the data should therefore have been analysed using a non-parametric test, such as the Mann–Whitney *U* test. The split between high and low responses to probiotic treatment observed in the original study was a potentially biologically interesting result, and the factors that determine a high-level response may be worth investigating, but this was not the conclusion that was reached by the authors.

A further issue arises from the way the results were presented in the study. Note that the y-axis spans 10 % in the original Fig. 1a, but only 5 % in the subsequent Fig. 1b summarizing the second experiment. The reader will intuitively judge the results on bar size, and be misled by a discrepancy in y-axis scaling, which effectively masks more substantial differences in response to the probiotic between the original and subsequent data sets. The difference between the probiotic and control treatments shown in Fig. 1b looks larger than it actually is, clearly shown when these data are displayed on the same scale as Fig. 1a (as shown in the modified Fig. 1d). Automated y-axis scaling to fit the data can lead to separate graphs with different scales, and this often has to be changed manually — something to be wary of when using automated graphing software.

